# Sex differences in adult asthma and COPD therapy: a systematic review

**DOI:** 10.1186/s12931-022-02140-4

**Published:** 2022-08-29

**Authors:** Paola Rogliani, Francesco Cavalli, Beatrice Ludovica Ritondo, Mario Cazzola, Luigino Calzetta

**Affiliations:** 1 Unit of Respiratory Medicine, Department of Experimental Medicine, University of Rome “Tor Vergata”, Via Montpellier, 1 – 00133 Rome, Italy; 2 Respiratory Medicine, University Hospital Policlinico Tor Vergata, Rome, Italy; 3 Department of Medicine and Surgery, Respiratory Disease and Lung Function Unit, University of Parma, Parma, Italy

**Keywords:** Asthma, COPD, Gender, Sex, Systematic review, Therapy

## Abstract

**Background:**

Although asthma is more prevalent in women and the prevalence of COPD is increasing in women, the current international recommendations for the management and prevention of asthma and COPD provide no sex-related indication for the treatment of these diseases. Therefore, we systematically reviewed the evidence across literature on the sex-related effectiveness of asthma and COPD therapy.

**Methods:**

This systematic review has been registered in PROSPERO and performed according to PRISMA-P. The PICO framework was applied for the literature search strategy: "patient problem” included adult patients suffering from asthma or COPD, “Intervention” regarded the pharmacological treatments for asthma or COPD, “Comparison” was vs. baseline, active controls, or placebo, “Outcome” was any difference sex-related in the effectiveness of interventions.

**Results:**

In asthma 44% of the evidence reported that men responded better than women to the therapy, whereas this percentage was 28% in COPD. ICS was generally less effective in women than in men to treat asthma, and consistent evidence suggests that in asthmatic patients ICS/LABA/LAMA combination may be equally effective in both men and women. Due to the inconsistent available evidence, it is not possible to identify specific treatments whose effectiveness is related to sex difference in COPD patients.

**Conclusions:**

There is a strong need of investigating the sex-related impact of asthma and COPD treatments. Pre-specified analyses in men and women should be planned in future trial protocols, a necessary condition that should be requested also by the regulatory agencies to overcome the anachronistic “one-size-fits-all” approach to therapeutics associated with suboptimal outcomes for patients.

## Background

Current data indicate that asthma and chronic obstructive pulmonary disease (COPD) affect together more than 600 million people worldwide and caused more than 3.5 million deaths per year [1–4]. The absolute number of patients suffering from asthma and COPD is increasing as the global population grows, and a relevant percentage of patients has been found to have suboptimal control of symptom burden [5].

Despite asthma is more prevalent in women and the prevalence of COPD is increasing in women [6, 7], and considering that cumulating evidence has highlighted the key pivotal role of sex differences in non-communicable diseases (NCDs) [8], the current international recommendations for the management and prevention of asthma and COPD [1, 2] do not provide any sex-related indication for the treatment of these diseases. Certainly, it may be also assumed that the lack of sex-specific recommendations for the treatment of asthma and COPD could be because no real difference in effectiveness exists but, unfortunately, to date it is not known whether this hypothesis is true [9]. In any case, it seems that both sex, assessed as male or female according to biological attributes, and gender, referred to social roles, behaviours, and expressions of identity, may significantly modulate the pharmacological response to asthma and COPD treatments [7, 10].

In this uncertain context, the aim of this article was to systematically review the evidence across literature on the sex-related effectiveness of pharmacotherapy in the treatment of asthma and COPD.

Indeed, a large body of evidence suggests that integrating data from randomized controlled trials (RCTs) and observational studies in systematic reviews and/or meta-analyses regarding complex interventions, such as the management of chronic obstructive pulmonary disorders according to the sex, may improve the prediction of patient responses to pharmacological therapies, resulting of high value and interest to patients, clinicians, policymakers, and other healthcare stakeholders [11, 12]. Moreover, including information also from observational studies may improve the inference based on RCTs [13]. Interestingly, these advantages of adding observational studies to RCTs to bring complementary healthcare information seems to be independent from the quality of the studies included [12]. Effectively, considering that it is unusual to find sufficient evidence from RCTs to answer all key questions in a systematic review, there is no a priori reason to exclude observational studies from a qualitative synthesis [13, 14]. After all, the greatest level in the new hierarchy of evidence is reached when both RCTs and observational studies exist with consistent findings [15].

Therefore, moving from this solid background and considering that the impact of sex differences in adult asthma and COPD therapy is a relevant but usually neglected topic, we carried out a systematic review by including both RCTs and observational studies.

## Methods

### Review question

The question of this systematic review was to assess sex-related differences in the effectiveness of pharmacological treatments for asthma and COPD.

### Search strategy

**Fig. 1:**
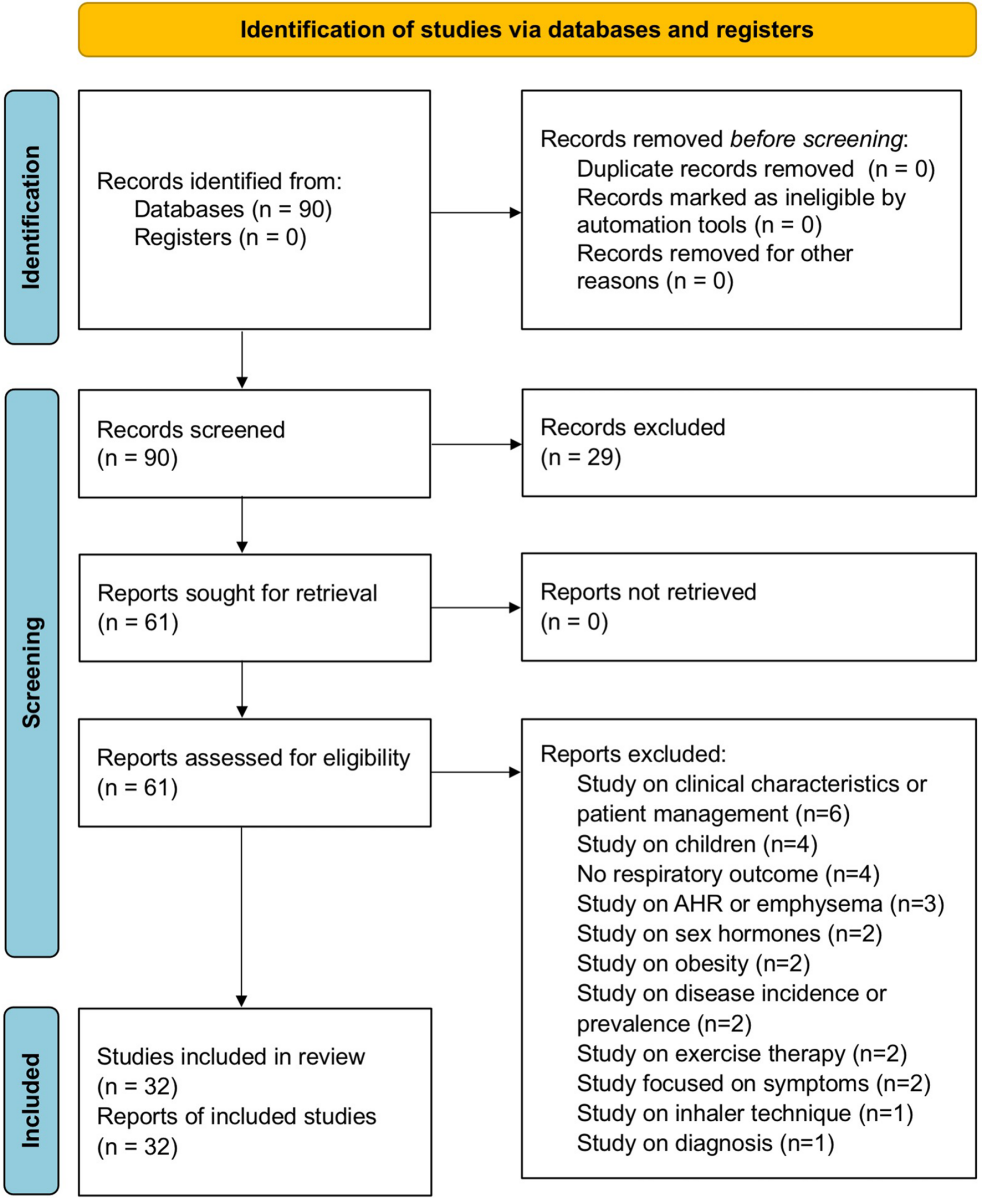
PRISMA 2020 flow diagram for the identification of the studies included in the systematic review. *AHR* airway hyperresponsiveness, *PRISMA* Preferred Reporting Items for Systematic Reviews and Meta-Analyses

This systematic review has been registered to the international prospective register of systematic reviews (PROSPERO, submission ID: 307060), and performed in agreement with the Preferred Reporting Items for Systematic Reviews and Meta-Analyses Protocols (PRISMA-P) [16]. The PRISMA 2020 flow diagram is shown in Fig. 1. This study satisfied all the recommended items reported by the PRISMA-P checklist [16]. A comprehensive literature search was performed for clinical trials assessing potential sex differences regarding the effectiveness of pharmacological treatments for asthma or COPD.

In this regard, the PICO (Patient problem, Intervention, Comparison, and Outcome) framework was applied to develop the literature search strategy, as previously reported [17]. Namely, the "patient problem” included adult patients suffering from asthma or COPD; the “intervention” regarded the administration of different pharmacological treatments for asthma or COPD; the “comparison” was performed with respect to baseline, active controls, or placebo (PCB); the assessed “outcome” was any difference related to sex in the effectiveness of pharmacological treatments for asthma and COPD.

The search was performed in ClinicalTrials.gov, Cochrane Central Register of Controlled Trials (CENTRAL), Embase, EU Clinical Trials Register, MEDLINE, Scopus, and Web of Science, in order to provide for relevant studies written in English and published up to January 3^rd^, 2022. The research string was as follows: (sex[Title] OR gender[Title]) AND (asthma OR COPD), “(("sex"[Title] OR "gender"[Title]) AND ("asthma"[MeSH Terms] OR "asthma"[All Fields] OR "asthmas"[All Fields] OR "asthma s"[All Fields] OR ("pulmonary disease, chronic obstructive"[MeSH Terms] OR ("pulmonary"[All Fields] AND "disease"[All Fields] AND "chronic"[All Fields] AND "obstructive"[All Fields]) OR "chronic obstructive pulmonary disease"[All Fields] OR "copd"[All Fields]))) AND (clinicaltrial[Filter] OR observationalstudy[Filter] OR randomizedcontrolledtrial[Filter])”. Citations of previous published reviews and commentaries were checked to select further pertinent studies, if any [6, 7, 18–22]. Literature search results were uploaded to Eppi-Reviewer 4 (EPPI-Centre Software. London, UK), a web-based software program for managing and analysing data in literature reviews that facilitates collaboration among reviewers during the study selection process.

### Study selection

Clinical trials that enrolled adult asthmatic or COPD patients and assessing sex-related differences in the effectiveness of pharmacological treatments for asthma or COPD were included in the systematic review. Two reviewers independently examined the studies, and any difference in opinion concerning the selection of relevant studies from literature searches and databases was resolved by consensus.

### Data extraction

Data from included clinical trials were extracted from published papers and/or supplementary files. Data were checked for study references and characteristics, number of analysed patients, treatments and comparators with doses of medications, regimen of administration, and type of inhaler, main inclusion criteria, age, sex, smoking habit, forced expiratory volume in the 1^st^ second (FEV_1_), exacerbation rate, any efficacy outcome measurements to detect potential differences between men and women, and study quality assessment via the Jadad Score [23], Cochrane Risk of Bias 2 (RoB 2) [24], Newcastle–Ottawa Scale (NOS) score [25], and Joanna Briggs Institute (JBI) Critical Appraisal Checklist Tool [26].

Data were extracted in agreement with Data Extraction for Complex Meta-anALysis (DECiMAL) recommendations [27].

### Endpoint

The endpoint of this systematic review was to assess sex-related differences in the effectiveness of pharmacological treatments used for asthma and COPD.

### Strategy for data synthesis

Data from original papers were extracted and reported via qualitative synthesis. Parts of Whole analysis via 10 × 10 dot plot graph was used to report the amount of evidence concerning the impact of sex on the response to the overall treatments in asthma and COPD. Bar Charts were used to show the response to pharmacological treatments in asthma and COPD according to specific outcomes and number of evidences.

### Quality of studies and risk bias

The summary of the risk of bias for each included randomized trial was analyzed via the Cochrane RoB 2 [24] and Jadad score [23]. The weighted assessment of the overall risk of bias was analyzed via the Cochrane RoB 2 [24] by using the robvis visualization software [28, 29].

The Jadad score, with a scale of 1–5 (score of 5 being the best quality), used to assess the quality of the clinical trials concerning the likelihood of bias related with randomization, double blinding, withdrawals, and dropouts. The quality of studies was assessed as follows: total score ≤ 2, low quality; total score = 3, medium quality; total score ≥ 4 high quality.

The NOS was used to assess the quality of observational cohort studies [25]. According to NOS, a study can be awarded with a maximum of one star for each item within the “Selection” and “Outcome” and a maximum of two stars can be given for “Comparability” [25]. In the present systematic review, the NOS quality assessment score was established to be in the range between zero and a maximum of nine stars. Studies reporting a NOS score ≥ 7 were considered of high quality, whereas those reporting a NOS score ≤ 6 were considered of low quality. For the NOS category “Outcome”, a follow-up period of at least ≃6 months was considered adequate to obtain the outcomes of interest from the included studies [30].

The methodological quality of observational cross-sectional studies was evaluated by using the JBI Critical Appraisal Checklist Tool for analytical cross-sectional studies [26]. The checklist consisted of eight question items assessing the inclusion criteria for the definition and detailed description of the sample, use of valid and reliable way to measure the exposure, use of objective and standard criteria to measure the condition, identification, and strategies to deal with confounding factors, use of a valid and reliable way to measure outcomes, and suitability of statistical analysis. In the present systematic review, each item of the JBI checklist was rated as “yes” and given 1 point and “no”, “unclear” or “not applicable” and given 0 points. The quality assessment score was calculated on the proportion of “yes” responses for the possible maximum score and judges at high risk, moderate risk or low risk of bias in agreement with the percentage of the achieved score, that was ≤ 49%, 50–69%, or ≥ 70%, respectively. Two reviewers independently assessed the quality of individual studies, and any difference in opinion about the quality score was resolved by consensus.

## Results

### Study characteristics

Of the 90 potentially relevant records identified in the initial search, 32 studies were deemed eligible for a qualitative synthesis (Table 1). This systematic review included data obtained from studies performed on patients with asthma [31–39], COPD [40–61], and populations in which both asthmatic and COPD patients were included [62].

**Table 1:** Main characteristics of the studies included in the systematic review

Study, year and reference	Number identifier	Study characteristics	Treatment duration (months)	Number of analyzed patients	Drugs, doses, and regimen of administration	Route of administration	Inhaler device (brand)	Patients’ characteristics
Nerpin et al. 2021 [36]	NA	Observational, multicenter, prospective, population-based cohort study based on the 3^rd^ European Community Respiratory Health Survey (ECRHS III)	1 day	651	SALB 200 μg single dose	Oral inhalation	MDI (NA)	Asthma (≥ 1 asthma-related symptom, including wheeze, nocturnal chest tightness or attacks of breathlessness following activity, at rest or at night and/or reported current use of ICSs in the previous year)
Harvey et al. 2020 [38]	ACTRN12618001497291	Observational, multicenter, prospective, post-marketing surveillance study based on the Australian Mepolizumab Registry (AMR)	12.0	309	Mepolizumab 100 mg Q4W	SC injection	/	Severe uncontrolled eosinophilic asthma (FEV_1_ ≤ 80% predicted; confirmed variable obstruction: 1) FEV_1_ reversibility ≥ 12% and ≥ 200 mL within 30 min after administration of salbutamol 200–400 μg or 2) AHR defined as > 20% decline in FEV_1_ during a direct bronchial provocation test or > 15% decline during an indirect test or 3) PEF variability of > 15% between the two highest and two lowest PEF rates during 14 days; ACQ-5 score ≥ 2 in the previous month and 1) ≥ 1 hospitalization for a severe asthma exacerbation or 2) ≥ 1 severe asthma exacerbation requiring use of OCSs initiated or increased for ≥ 3 days or parenteral corticosteroids prescribed/supervised by a physician)
Ohar et al. 2020 [45]	NCT02347761 (GOLDEN 3), NCT02347774 (GOLDEN 4)	Pooled analysis of 2 replicate Phase III, multicenter, randomized, double-blind, PCB-controlled, parallel group	3.0	861	GLY 25 μg BID vs. PCB	Oral inhalation	eFlow® nebulizer	Moderate to severe COPD (post-bronchodilator FEV_1_ ≤ 80% predicted and FEV_1_/FVC < 0.7)
Colombo et al. 2019 [37]	NA	Post-hoc analysis of the observational, multicenter, non-controlled, cohort PROXIMA study including a cross-sectional and prospective longitudinal phases (omalizumab was administered exclusively in the longitudinal phase)	12.0	99 (in the longitudinal phase)	Add-on omalizumab 75—600 mg Q4W	SC injection	/	Severe allergic asthma
D’Urzo et al. 2019 [51]	NCT01462942 (ACLIFORM), NCT01437397 (AUGMENT)	Pooled analysis of 2 Phase III, multicenter, randomized, double-blind, active-and PCB-controlled, parallel group studies	24.0	2684	ACL/FOR 400/12 μg BID vs. ACL 400 μg vs. FOR 12 μg vs. PCB	Oral inhalation	DPI (Genuair™/Pressair®)	Moderate to severe stable COPD (post-bronchodilator FEV_1_ ≥ 30% and < 80% predicted and FEV_1_/FVC < 0.7)
Wedzicha et al. 2019 [52]	NCT01782326 (FLAME)	Post-hoc analysis of the randomized, double-blind, double-dummy, active-controlled, parallel group FLAME trial	12.0	3362	IND/GLY 110/50 μg QD vs. FP/SAL 50/500 μg BID	Oral inhalation	IND/GLY: DPI (Breezhaler®); FP/SAL: DPI (Accuhaler®)	Moderate to severe COPD (post-bronchodilator FEV_1_ ≥ 25% and < 60% predicted and FEV_1_/FVC < 0.7; ≥ 1 exacerbation in the previous year)
Martinez et al. 2018 [54]	NCT01329029 (REACT), NCT01443845 (RE2SPOND)	Pooled analysis of 2 Phase IV, multicenter, randomized, double-blind, PCB-controlled, parallel group studies	12.0	4287	Add-on roflumilast 500 μg QD vs. PCB	PO	/	Severe or very severe COPD (post-bronchodilator FEV < 50% predicted and FEV_1_/FVC < 0.7)
Li et al. 2017 [44]	NA	Post-hoc analysis of the multicenter, randomized, PCB-controlled, parallel-group LHS study	60.0	5887	IB 72 μg TID vs. PCB	Oral inhalation	NA	Mild to moderate COPD (post-bronchodilator FEV_1_ ≥ 55% and ≤ 90% predicted and FEV_1_/FVC < 0.7)
Tsiligianni et al. 2017 [50]	NCT01120717 (ENLIGHTEN), NCT01202188 (SHINE), NCT01120691 (SPARK), NCT01315249 (ILLUMINATE), NCT01285492 (ARISE), NCT01709903 (LANTERN)	Pooled analysis of 6 randomized, PCB- or active-controlled, parallel group studies (data from the ARISE on Japanese population only)	26.0–64.0	6108	IND/GLY 100/50 μg QD vs. FP/SAL 500/50 μg BID vs. GLY 50 μg QD vs. TIO 18 μg QD vs. PCB	Oral inhalation	IND/GLY: DPI (Breezhaler®); FP/SAL: DPI (Accuhaler®)	Moderate to severe COPD or severe to very severe COPD in the SPARK (post-bronchodilator FEV_1_ ≥ 30% and < 80% predicted [except SPARK where patients were having post-bronchodilator < 50% predicted], and a FEV_1_/FVC < 0.7; history of ≤ 1 exacerbation at baseline for inclusion into the LANTERN and a history of ≥ 1 exacerbation in the previous year for inclusion in the SPARK)
Kerstjens et al. 2016, PrimoTinAasthma® [34]	NCT00772538, NCT00776984	Two Phase III, randomized, double-blind, PCB-controlled, parallel group	48.0	912	Add-on TIO 5 μg QD vs. PCB to ICS/LABA	Oral inhalation	SMI (Respimat®)	Severe symptomatic asthma (post-bronchodilator FEV_1_ ≤ 80% predicted and FEV_1_/FVC ≤ 0.7 measured 30 min after inhaling 400 μg of salbutamol at screening; daily treatment with ≥ 800 μg of BUD or equivalent dose of another ICS + LABA for ≥ 4 weeks before screening; ≥ 1 exacerbation requiring treatment with SCSs in the previous year; ACQ-7 score ≥ 1.5)
Han et al. 2014 [56]	NA	Subgroup analysis of a multicenter, prospective, randomized, double-blind, PCB-controlled, parallel group study (NCT00325897)	12.0	1113	Azithromycin 250 mg QD vs. PCB	PO	/	COPD (post-bronchodilator FEV_1_ < 80% predicted and FEV_1_/FVC < 0.7)
Yu et al. 2014 [53]	NA	Analysis of trial data released by the US FDA	≤ 12.0	NA	Add-on roflumilast 500 μg QD vs. PCB	PO	/	Moderate to severe COPD
Albert et al. 2011 [55]	NCT00325897	Multicenter, prospective, randomized, double-blind, PCB-controlled, parallel group	12.0	1142	Azithromycin 250 mg QD vs. PCB	PO	/	COPD (post-bronchodilator FEV_1_ < 80% predicted and FEV_1_/FVC < 0.7)
Celli et al. 2011 [61]	NCT00268216 (TORCH)	Extended-analysis of the Phase III, randomized, double-blind, PCB-controlled, parallel group TORCH study	36.0	6112	FP 500 μg BID vs. SAL 50 μg BID vs. PCB	Oral inhalation	DPI (Accuhaler®)	COPD (pre-bronchodilator FEV_1_ < 60% predicted and pre-bronchodilator FEV_1_/FVC ≤ 0.7; reversibility of FEV_1_ to 400 μg salbutamol of < 10% of predicted)
Tashkin et al. 2011 [60]	NCT00285012	Multicenter, Phase III, randomized, double-blind, PCB-controlled, parallel group	3.0	499	Varenicline 0.5 mg QD for 3 days, 0.5 mg BID for 4 days, then 1 mg BID until end of the study	PO	/	Mild to moderate COPD (post-bronchodilator FEV_1_ ≥ 50% and FEV_1_/FVC < 0.7)
Tashkin et al. 2011 [49]	NA	Post-hoc analysis of a multicenter, randomized, double-blind, active-controlled, parallel group study	3.0	255	FOR 12 μg BID + TIO 18 μg QD vs. TIO 18 μg QD	Oral inhalation	DPI (NA)	COPD (post-bronchodilator FEV_1_ > 30% and < 70% predicted and FEV_1_/FVC < 0.7)
Lopez-Varela et al. 2010, PLATINO [58]	NA	Multicenter, cross-sectional, population-based survey	1 day	759	SALB 200 μg single dose	Oral inhalation	NA	COPD
Tashkin et al. 2010 [43]	NCT00144339 (UPLIFT)	Subgroup analysis of the Phase III, multicenter, randomized, double-blind, PCB-controlled, parallel group UPLIFT study	48.0	5992	TIO 18 μg QD vs. PCB	Oral inhalation	DPI (HandiHaler®)	COPD (post-bronchodilator FEV_1_ < 70% predicted and FEV_1_/FVC ≤ 0.7)
Siroux et al. 2009 [32]	NA	Observational, cross-sectional study using data from the case–control and family-based EGEA2 study	12.0	501	ICS	Oral inhalation	NA	Current asthma
Celli et al. 2008 [48]	NCT00268216 (TORCH)	Post-hoc analysis of the Phase III, randomized, double-blind, PCB-controlled, parallel group TORCH study	36.0	5,343	FP/SAL 500/50 μg BID vs. FP 500 μg BID vs. SAL 50 μg BID vs. PCB	Oral inhalation	DPI (Accuhaler®)	COPD (pre-bronchodilator FEV_1_ < 60% predicted and pre-bronchodilator FEV_1_/FVC ≤ 0.7; reversibility of FEV_1_ to 400 μg salbutamol of < 10% of predicted)
Calverley et al. 2007, TORCH [46]	NCT00268216	Phase III, randomized, double-blind, PCB-controlled, parallel group	36.0	6112	FP/SAL 500/50 μg BID vs. FP 500 μg BID vs. SAL 50 μg BID vs. PCB	Oral inhalation	DPI (Accuhaler®)	COPD (pre-bronchodilator FEV_1_ < 60% predicted and pre-bronchodilator FEV_1_/FVC ≤ 0.7; reversibility of FEV_1_ to 400 μg salbutamol of < 10% of predicted)
Soriano et al. 2007, ISEEC study [42]	NA	Pooled analysis of 7 randomized, double-blind, PCB-controlled, parallel group studies	12.0–36.0	3911	ICS use (triamcinolone 1200 μg QD; BUD 800—867 μg QD; FP 1000 μg QD)	Oral inhalation	NA	Moderate to severe COPD
Dales et al. 2006 [62]	NA	Observational study in primary-care settings	1 day	187	SALB 200 μg single dose	Oral inhalation	NA	COPD and asthma
Dijkastra et al. 2006 [33]	NA	Observational, retrospective, cohort study	23 years of follow-up	122	ICS vs. no ICS use	Oral inhalation	NA	Moderate to severe asthma
Watson et al. 2006 [41]	NA	Post-hoc analysis of the multicenter, randomized, double-blind, PCB-controlled, parallel group EUROSCOP study	36.0	1128	BUD 400 μg BID vs. PCB	Oral inhalation	DPI (Turbuhaler®)	Mild to moderate COPD (post-bronchodilator FEV_1_ 50–100% and FEV_1_/FVC < 0.7; < 10% predicted increase in FEV_1_ after inhalation of 1 mg terbutaline)
Anthonisen et al. 2005 [59]	NA	Post-hoc analysis of a selected cohort from the multicenter, randomized, PCB-controlled, parallel-group LHS study	11 years	4194	Isoproterenol 200 μg	Oral inhalation	MDI (NA)	COPD (post-bronchodilator FEV_1_ ≥ 55 and ≤ 90% predicted and FEV_1_/FVC < 0.7)
Bousquet et al. 2005 [39]	NA	Pooled analysis of 7 randomized, double-blind, PCB-controlled, parallel-group studies and two randomized, open-label, active-controlled, parallel group studies	5.5–12.0	4308	Add-on omalizumab at least 0.016 mg/kg per IU/mL of IgE Q2W or Q4W vs. PCB or current asthma therapy without omalizumab	SC injection	/	Severe persistent asthma
Schermer et al. 2004 [40]	NA	Prospective, clinical-practice setting, unblinded study of the ICS washout phase of the COOPT trial	3.0	201	ICS discontinuation (FP, BUD, or BDP)	Oral inhalation	NA	COPD (post-bronchodilator FEV_1_ < 90% predicted or FEV_1_/FVC < 0.88 [< 0.89 for women])
Vestbo et al. 2004 [47]	NA	Sensitivity analysis of the multicenter, randomized, double-blind, PCB-controlled, parallel group TRISTAN study	12.0	719	FP/SAL 500/50 μg BID vs. PCB	Oral inhalation	DPI (Advair Diskus®)	COPD (pre-bronchodilator FEV_1_ ≥ 25 and ≤ 70% predicted and FEV_1_/FVC < 0.7; reversibility < 10% predicted FEV_1_)
Convery et al. 2000 [31]	NA	Randomized, double-blind PCB-controlled, parallel group	1.5	52	FP 2000 μg QD vs. PCB	Oral inhalation	pMDI (NA)	Mild asthma (treatment-naïve patients)
Lima et al. 2000 [35]	NA	PD study	1 day	30	SALB 8 mg single dose	PO	/	Moderate asthma
Kanner et al. 1994 [57]	NA	Multicenter, randomized, PCB-controlled, parallel-group	60.0	5662	Smoking cessation + IB vs. smoking cessations + PCB	IB: oral inhalation	NA	COPD (post-bronchodilator FEV_1_ ≥ 55% and ≤ 90% predicted and FEV_1_/FVC < 0.7)

Age (years)	Male (%)	Current smokers (%)	Post bronchodilator FEV_1_ (% predicted)	Evaluated outcomes	Jadad score	NOS Quality Assessment				JBI checklist Tool
						**Selection**	**Comparability**	**Outcome**	**Total**	
53.7	39.3	14.9	87.3	Lung function	/	***	NA	**	5	/
59.6	42.4	0.6	62.7	Disease control	/	****	NA	***	7	/
63.5	55.4	NA	52.5	Lung function, exacerbations, QoL, and rescue medication use	All RCTs: 5	/	/	/	/	/
51.7	37.4	5.1	NA	Disease control, disease perception, and QoL	/	****	NA	**	6	/
63.6	60.0	49.2	NA	Lung function, exacerbations, and dyspnea	AUGMENT: 4, ACLIFORM: 5	/	/	/	/	/
63.9	76.0	42.6	44.7	Exacerbations, lung function, QoL, rescue medication use	FLAME: 5	/	/	/	/	/
64.6	71.3	41.3	34.11	Exacerbations	REACT: 5; RE^2^SPOND: 4	/	/	/	/	/
48.5	62.9	100.0	NA	Lung function	LHS: 3	/	/	/	/	/
63.1	77.2	41.6	NA	Lung function, QoL, rescue medication use	All RCTs: 5	/	/	/	/	/
53.0	39.6	0.0	NA	Lung function, exacerbations, disease worsening	PrimoTinAasthma®: 5	/	/	/	/	/
65.5	59.0	22.0	39.5	Exacerbations	NCT00325897: 4	/	/	/	/	/
NA	NA	NA	NA	Benefit-harm index	NA	/	/	/	/	/
65.5	59.0	22.0	39.5	Exacerbations	4	/	/	/	/	/
65.0	75.8	43.0	44.3	Exacerbations, QoL, and mortality	TORCH: 5	/	/	/	/	/
57.2	62.4	100.0	70.0	Smoking cessation	3	/	/	/	/	/
≥ 40.0	66.5	47.5	NA	Lung function	RCT: 5	/	/	/	/	/
64.2	52.3	36.0	NA	Acute bronchoreversibility	/	/	/	/	/	High bias
63.8	74.6	32.0	48.0	Lung function, exacerbations, QoL, and mortality	UPLIFT: 5	/	/	/	/	/
39.2	50.9	26.3	94.9	Lung function, exacerbations, disease symptoms	/					Low bias
64.9	76.3	44.3	44.7	Lung function	TORCH: 5	/	/	/	/	/
65.0	75.8	43.0	44.3	Mortality	5	/	/	/	/	/
58.3	70.8	73.0	60.3	Lung function	7 RCTs: between 3 and 5	/	/	/	/	/
59.0	38.0	NA	NA	Bronchoreversibility	/	/	/	/	/	Moderate bias
28.0	58.0	45.0	85.0	Lung function	/	****	NA	**	6	/
52.5	72.3	100.0	80.2	Disease symptoms	EUROSCOP: 3	/	/	/	/	/
50.1	61.9	25.5	78.5	Bronchoreversibility	LHS: 3	/	/	/	/	/
41.3	40.8	NA	70.2	Exacerbations	7 RCTs: between 2 and 3	/	/	/	/	/
60.6	68.0	49.0	65.6	Adverse respiratory outcome	/	****	NA	***	7	/
62.5	75.0	51.5	NA	Lung function, exacerbations, QoL	TRISTAN: 5	/	/	/	/	/
32.5	59.6	48.1	101.2	Lung function	2	/	/	/	/	/
18.0–50.0	53.3	0.0	92.8	Lung function	NA	/	/	/	/	/
48.5	62.8	100.0	75.0	Lung function	3	/	/	/	/	/

*indicates one star given to the “Selection”, “Comparability”, and “Outcome” categories according to the star-based NOS scoring system employed to assess the quality of each observational study, as detailed in the section “Quality of studies and risk of bias”. /: data not evaluable; *ACL* aclidinium, *ACQ* Asthma Control Questionnaire, *AHR* airway hyperresponsiveness, *BDP* beclomethasone dipropionate, *BID* bis in die, twice-daily, *BUD* budesonide; COPD: chronic obstructive pulmonary disease, *DPI* dry powder inhaler; *FDA* Food and Drug Administration, *FEV*_*1*_ forced expiratory volume in the 1st second, *FP* fluticasone propionate, *FOR* formoterol, *FVC* forced vital capacity, *GLY* glycopyrronium, *IB* ipratropium bromide; ICS: inhaled corticosteroid, *IND* indacaterol, *JBI* Joanna Briggs Institute, *MDI* metered dose inhaler, *NA* not available, *NOS* Newcastle–Ottawa Scale, *OCS* oral corticosteroid, *PC*_*20*_ Provocative concentration of methacholine causing a 20% fall in FEV_1_; *PCB* placebo, *PD* pharmacodynamics, *PEF* peak expiratory flow, *PK* pharmacokinetic: pMDI: pressurized metered dose inhaler; *PO* oral administration, *Q2W* once every 2 weeks; *Q4W* once every 4 weeks, *QD* quaque die, once-daily; *QoL* quality of life; *SAL* salmeterol, *SALB* salbutamol, *SC* subcutaneous, *SMI* soft mist inhaler; *TIO* tiotropium bromide

Overall, 6 studies [31, 34, 46, 55, 57, 60] RCTs, 4 studies [32, 33, 58, 62] were retrospective observational, 3 studies [36, 38, 40] were prospective observational, and 1 study [35] was focused on pharmacodynamics (PD). Eight [41, 44, 48, 49, 52, 59] studies were post-hoc analyses of RCTs and another one [37] of an observational study, 6 studies [39, 42, 45, 50, 51, 54] were pooled analyses of RCTs**,** 2 studies [43, 56] were subgroup analyses of RCTs, 1 study [61] was an extended analysis of a RCT, and 1 study [47] was a sensitivity analysis of a RCT. One study [53] reported an analysis of trial data released by the US Food and Drug Administration (FDA).

Tables 2 and 3 summarize the results of the studies in which a sex-related difference in the effectiveness of asthma and COPD therapies has been assessed.

**Table 2:** Evidence from the studies included in the systematic review concerning the sex-related differences in the effectiveness of asthma treatments

Outcomes	Treatments and comparisons				
	ICS	SABA	ICS/LABA/LAMA	Omalizumab	Mepolizumab
	vs. PCB or baseline	vs. baseline	vs. ICS/LABA	vs. PCB or baseline	vs. PCB
FEV_1_	[33]: men responded significantly better than women	[35, 36]: women ≈ men	[34]: women ≈ men	/	/
FEV_1_/FVC	/	[36]: women responded significantly better than men	/	/	/
Protection against bronchial provocation	[31]: men responded significantly better than women	/	/	/	/
Exacerbation	[32]: borderdline significance only in men	/	[34]: women ≈ men	[39]: women ≈ men	/
Time to first episode of asthma worsening	/	/	[34]: women ≈ men	/	/
Asthma control	[32]: men responded significantly better than women	/	/	[37]: women ≈ men	[38]: women responded significantly better than men
Asthma symptoms	[32]: men responded significantly better than women	/	/	/	/
Asthma perception	/	/	/	[37]: men responded significantly better than women	/
Quality of life	/	/	/	[37]: men responded significantly better than women	/
FeNO	/	[36]: significantly greater in women than men	/	/	/

The greater response of a gender vs. the other one was reported when a statistically significant (P < 0.05) superiority was detected in the reference study for a specific treatment; the symbol “≈” indicates a similar, not statistically different (P ≥ 0.05) response between women and men to a specific treatment

/: data not available, *FeNO* fraction exhaled nitric oxide, *FEV*_*1*_ forced expiratory volume in the 1st second, *FVC* forced vital capacity, *ICS* inhaled corticosteroid, *LABA* long-acting β_2_-adrenoceptor agonist, *LAMA* long-acting muscarinic antagonist, *PCB* placebo

**Table 3:** Evidence from the studies included in the systematic review concerning the sex-related differences in the effectiveness of COPD treatments

Outcomes	Treatments and comparisons											
	Muscarinic antagonists	ICS		Short-acting bronchodilators	LABA/LAMA				ICS/LABA	PDE4 inhibitor	Azithromycin	Varenicline
	vs. PCB or baseline	vs. PCB or baseline	Discontinuation	vs. baseline	vs. LAMA	vs. LABA	vs. ICS/LABA	vs. PCB	vs. PCB	vs. PCB	vs. PCB	vs. PCB
FEV_1_	[43, 45]**:** women ≈ men; [44]: women responded significantly better than men	[42]: women ≈ men	/	[58]: women responded significantly better than men; [59]: men responded significantly better than women	[49]: women ≈ men; [50]: women responded significantly better than men; [51]: men responded significantly better than women	[51]: women ≈ men	[50]: men responded significantly better than women; [52]: women ≈ men	[50]: men responded significantly better than women; [51]: women ≈ men	[47, 48]: women ≈ men	/	/	/
Protection against bronchial provocation	/	/	/	[57]: numerically better in women than in men	/	/	/	/	/	/	/	/
Exacerbation	[43]**:** women ≈ men	/	/	/	[51]: women ≈ men	[51]: women ≈ men	[52]: significant only in men (numerical improvement in women)	[51]: women ≈ men	[61]: men responded significantly better than women; [47]: women ≈ men	[54] significant only in men (numerical improvement in women)	[55, 56]: women ≈ men	/
EXACT or EXACT-RS	[45]: significant reduction in women but not in men	/	/	/	/	/	/	[51]: significant only in men	/	/	/	/
Adverse respiratory outcome	/	/	[40]: men responded significantly better than women	/	/	/	/	/		/	/	/
TDI	/	/	/	/	[50]: women responded significantly better than men	[51]: significant only in men	[50]: women responded significantly better than men	[50]: women responded significantly better than men; [51]: women ≈ men	/	/	/	/
Symptom total score	/	/	/	/	[50]: women responded significantly better than men	/	[50]: women responded significantly better than men	[50]: women responded significantly better than men	/	/	/	/
Phlegm symptoms	/	[41]: significant reduction in men but not in women	/	/	/	/	/	/		/	/	/
Wheeze, dyspnea, and cough symptoms	/	[41]: women ≈ men	/	/	/	/	/	/		/	/	/
Rescue medication	[45]**:** women ≈ men	/	/	/	[50]: numerically better in women than in men	/	[50]: numerically better in women than in men; [52]: significant only in men (numerical improvement in women)	[50]: numerically better in women than in men	/	/	/	/
SGRQ	[43]**,** [45]**:** women ≈ men	/	/	/	[50]: women responded significantly better than men	/	[50]: women better than men; [52]: significant only in men (numerical improvement in women)	[50]: women responded significantly better than men	[61]: women ≈ men; [47]: significant only in men (numerical improvement in women)	/	/	/
Mortality	[43]**:** significant only in men (numerical improvement in women)	/	/	/	/	/	/	/	[46]: women ≈ men; [61]: numerically better in women than in men	/	/	/
Smoking cessation	/	/	/	/	/	/	/	/	/	/	/	[60]: women ≈ men

The greater response of a gender vs. the other one was reported when a statistically significant (P < 0.05) superiority was detected in the reference study for a specific treatment; the symbol “≈” indicates a similar, not statistically different (P ≥ 0.05) response between women and men to a specific treatment

/: data not available, *EXACT* Exacerbation of Chronic Pulmonary Disease Tool, *EXACT-RS* EXACT-respiratory symptoms, *FEV*_*1*_ forced expiratory volume in the 1st second, *FVC* forced vital capacity, *ICS* inhaled corticosteroid, *LABA* long-acting β_2_-adrenoceptor agonist, *LAMA* long-acting muscarinic antagonist, *PCB* placebo, *PDE4* phosphodiesterase 4, *SGRQ* St George's Respiratory Questionnaire, *TDI* Transition Dyspnea Index

### Sex differences in asthma therapy

#### ICS

Intermittent pulsed therapy at 2 week-intervals with fluticasone propionate (FP) 2000 μg once daily (QD) for 6 weeks induced a short-term benefit on airway responsiveness that was lower in treatment-naïve women than in men with mild asthma, by producing respectively 1.2 vs. 3.2 doublings in the provocative dose of methacholine causing a 20% fall in FEV_1_ (PD_20_) (P < 0.05) [31].

In a cross-sectional French study on asthmatic patients [32], women treated with inhaled corticosteroids (ICS) in the past year were at significant (P < 0.05) greater risk for uncontrolled asthma than men. Men treated with ICS showed a borderline significant reduction in the risk for severe exacerbation (P = 0.05) and had a lower frequency of symptoms than women (odds ratio [OR] 0.30, 95% confidence interval [95%CI] 0.15–0.59; P < 0.001) [32].

In moderate to severe asthmatic men, treatment with ICS over a period of 23 years reduced the annual decline in FEV_1_ of 20.6 mL/year compared to the time before starting with ICS (P < 0.05), but this effect was not observed in women [33]. ICS use induced an improvement of 36.8 mL/year in the annual decline of FEV_1_ only in men smoking < 5 pack/years (P < 0.01) and the difference between sexes was significant (P < 0.05) [33]. In patients smoking ≥ 5 pack/years, no change in the decline of FEV_1_ was observed in both men and women [33]. A greater daily ICS dose was associated with a minor decline in FEV_1_ in men (P < 0.01), an effect not observed in women [33].

#### SABA

In a study focusing on the PD response to a single-dose of salbutamol (SALB) 8 mg administered to moderate asthmatic patients [35], salbutamol increased FEV_1_ from baseline in men (+ 620 mL, range 110–3300; P < 0.05) and women (+ 310 mL, range 100–770; P < 0.05), as well as FEV_1_% predicted in men (13.5%, range 1–76; P < 0.05) and women (12%, range 4–24; P < 0.05) [35]. The mean plasma concentration of SALB at which maximal bronchodilation was evoked was numerically greater in men than women.

#### ICS/LABA/LAMA

In two RCTs [34] conducted in parallel in patients with severe symptomatic asthma and treated with the add-on long-acting muscarinic antagonist (LAMA) tiotropium (TIO) 5 μg QD to ICS plus a long-acting β_2_-adrenoceptor (β_2_-AR) agonist (LABA), sex did not exert an influence on the improvement in peak FEV_1_, in the time to first severe asthma exacerbation, and in the time to first episode of asthma worsening vs. ICS/LABA.

#### Bronchoreversibility to short acting bronchodilators

In a recent analysis of data from the third European Community Respiratory Health Survey (ECRHS III) [36], the bronchodilator (BD) response to SALB 200 μg with regards to FEV_1_/forced vital capacity (FVC) was superior in asthmatic women than in men (4.1, 95%CI 3.6–4.6 vs. 3.0, 95%CI 2.5–3.6; P < 0.01 vs. pre-BD). The BD response with respect to FEV_1_ was improved in both men (4.9, 95%CI 4.1–5.8; P < 0.05 vs. pre-BD) and women (5.0, 95%CI 4.2–5.7; P < 0.05 vs. pre-BD). The increase in FEV_1_ was positively associated with the fraction exhaled of nitric oxide levels after BD use in women (P < 0.05), whereas men showed no difference [36].

#### Monoclonal antibodies

Mepolizumab is an anti-interleukin-5 monoclonal antibody (mAb) approved for the treatment of severe eosinophilic asthma [63]. A recent real-world observational study of the post-marketing surveillance Australian Mepolizumab (MEPO) Registry [38] found that after treatment with mepolizumab, a greater number of women than men with severe eosinophilic asthma were classified as Asthma Control Questionnaire (ACQ) super-responders (67.0 vs. 43.0%; P < 0.01), meaning that women were more likely to achieve the best control over asthma symptoms with mepolizumab.

Omalizumab is a humanized mAb that blocks the interaction between IgE and high-affinity receptor FcεRI on inflammatory cells; it is approved for the treatment of patients with persistent severe allergic asthma, high levels of blood IgE, and at least a sensitization to a perennial allergen [63]. In a post-hoc analysis of the Patient Reported Outcomes and Xolair® In the Management of Asthma (PROXIMA) study [37], one year of treatment with omalizumab improved median ACQ scores from baseline in men (1.1 units, 95%CI 0.4–1.7; P < 0.05) and women (1.4 units, 95%CI 1.0–2.4; P < 0.05), and the asthma control rates were similar by sex. Asthma perception was worse in women than men, reaching Brief Illness Perception Questionnaire (B-IPQ) total scores of 41.8 ± 9.4 and 35.6 ± 12.0 units, respectively (P < 0.05) [37]. Sex-related differences were observed for some specific items of the B-IPQ score, with men reporting less asthma symptoms than women (4.8 ± 2.5 vs. 5.9 ± 2.4 units), less concern about the disease (4.9 ± 2.7 vs. 6.1 ± 2.8 units), lower emotional impact by the illness (4.6 ± 2.6 vs. 6.2 ± 2.7 units), and greater control by the treatment (8.7 ± 1.4 vs. 8.0 ± 2.0 units) (P < 0.05) [37]. Men had a better health status than women, reporting an EuroQoL score of 0.93 vs. 0.86 units at 12 months of therapy [37].

In a pooled analysis of data from 7 RCTs [39], treatment every 2 or 4 weeks with add-on omalizumab similarly reduced the annualized exacerbation rate in men (RR 0.67, 95%CI 0.51–0.76; P < 0.0001 vs. PCB) and women (RR 0.61, 95%CI 0.52–0.72; P < 0.0001 vs. PCB) affected by severe persistent asthma.

### Sex differences in COPD therapy

#### ICS

According to a prospective unblinded study [40] conducted in primary care settings, women suffering from COPD who discontinued treatment with an ICS were at significantly higher risk of an adverse respiratory outcome than men (hazard ratio [HR] 2.14, 95%CI 1.31–3.50; P < 0.01).

A post-hoc analysis of the European Respiratory Society Study on Chronic Obstructive Pulmonary Disease (EUROSCOP) [41] reported that 3 years of treatment with budesonide (BUD) 400 μg BID reduced the prevalence of phlegm symptoms (OR 0.66, 95%CI 0.52–0.83; P < 0.05 vs. PCB) in men but not in women. No change in the prevalence of wheeze, dyspnoea, and cough symptoms was detected after treatment, irrespective of sex [41].

The Inhaled Steroids Effect Evaluation in COPD (ISEEC) pooled analysis [42] of seven RCTs assessing the effectiveness of long-term ICS use in moderate to severe COPD, indicated that over the first 6 months of treatment, ICSs improved FEV_1_ in both men (+ 42 mL) and women (+ 29 mL) compared to PCB (P < 0.01). In the ex-smoker group, women had a larger increase in FEV_1_ with ICS therapy than did men [42]. From 6 to 36 months of therapy, both men and women from the ICS group had a similar and significant (P < 0.05) decrease in FEV_1_ from baseline of -25 mL and -24 mL, respectively [42].

#### Muscarinic antagonists

In a subgroup analysis of the 4-year Understanding the Potential Long-term Impact of Tiotropium (UPLIFT) RCT [43], TIO 18 μg QD improved trough FEV_1_ in both men and women (92 mL and 77 mL, respectively; P < 0.001 vs. PCB), although the annualized rates of decline in predicted FEV_1_ were similar to PCB and by sex. TIO reduced the risk for a first exacerbation in men (HR 0.87, 95%CI 0.81–0.93; P < 0.05) and women (HR 0.83, 95%CI 0.74–0.94; P < 0.05) compared to PCB, as well as the number of exacerbations per patient-year in men (from 0.82 ± 0.02 to 0.71 ± 0.02; P < 0.005) and in women (from 0.92 ± 0.04 to 0.77 ± 0.03; P < 0.005) [43]. TIO lowered the risk of all-cause mortality irrespective of sex, although the effect was significant (P < 0.05) only in men (HR 0.85, 95%CI 0.72–0.99) [43]. Total St George's Respiratory Questionnaire (SGRQ) scores were improved with TIO in both men (between -2.3 and -3.6 units; P < 0.05 vs. PCB) and women (between -2.1 and -2.7 units; P < 0.05 vs. PCB) [43].

Li et al. [44] used data from the Lung Health Study (LHS) to investigate sex-related differences in BD response following treatment with ipratropium bromide (IB) administered at 72 μg three times a day, in mild to moderate COPD patients. After 4 months, IB improved FEV_1_ from baseline by 2.94 ± 7.53% in men and by 6.0 ± 7.51% in women, a sex-related difference that persisted for 2 years (P < 0.05), but beyond this time point, the greater beneficial impact on FEV_1_ in women was lost [44]. The BD effect of IB was found to be inversely related with body mass index (BMI), therefore women in the lowest BMI categories experienced greater benefits from therapy (P < 0.05), whereas BMI had no impact on the pharmacological response in men [44].

A pooled analysis [45] of data from moderate to severe COPD patients who participated in the GOLDEN 3 and GOLDEN 4 replicate studies found that 12 weeks of treatment with glycopyrronium bromide (GLY) 25 μg BID improved trough FEV_1_ in both men (+ 86 mL) and women (+ 102 mL) (P < 0.001 vs. PCB). GLY was superior to PCB in reducing SGRQ scores in men (-3.19 units) and women (-3.58 units) (P < 0.01), with no difference by sex [45]. Although the Exacerbation of Chronic Pulmonary Disease Tool (EXACT)-respiratory symptoms (EXACT-RS) total score was reduced regardless of sex with GLY, only women achieved a significant (P < 0.01) improvement compared to PCB (-1.48 units) and to men (-2.33 units) [45]. Changes in rescue medication use were not different across treatment groups and by sex [45].

#### ICS/LABA and LABA/LAMA

In the Toward a Revolution in COPD Health (TORCH) study [46], a RCT primarily designed to determine the mortality risk from any cause over 3 years of treatment with FP/salmeterol (FP/SAL) 500/50 μg BID and its monocomponents, sex-related differences did not affect any treatment response vs. PCB. An extended analysis of the TORCH RCT [61] found that over 3 years of study, women had a numerically lower risk of mortality than men. The rate of exacerbations was higher by 25.0% (95% CI 16–34; P < 0.001) in women than in men but no difference by sex was observed in the change of SGRQ [61]. According to a post-hoc analysis of the TORCH RCT [48], the treatment effect of FP/SAL 500/50 μg BID combination or its monocomponents on the rate of FEV_1_ decline was similar irrespective of sex.

In a sensitivity analysis of The TRial of Inhaled STeroids ANd long-acting β_2_ agonists (TRISTAN) RCT [47], 1 year of treatment with FP/SAL 500/50 μg BID improved pre-treatment FEV_1_ in both men (+ 127 mL, 95%CI 94–159; P < 0.05 vs. PCB) and women with COPD (+ 152 mL, 95%CI 95–208; P < 0.05; vs. PCB). FP/SAL reduced the rate of COPD exacerbations in men by 23.0% (95%CI 8.0–35.0; P < 0.01) and in women by 31.0% (95%CI 9.0–48.0; P < 0.01) compared to PCB; the rate of severe COPD exacerbations was decreased respectively in men by 41.0% (95%CI 25.0–53.0; P < 0.001) and in women by 36.0% (95%CI 9.0–55.0; P < 0.05) [47]. Combination therapy induced a significant (P < 0.05) improvement in SGRQ scores in men (-2.1 units, 95%CI -3.5 – -0.8) and a numerical decrease in women [47].

In a post-hoc analysis [49] of a 12-week RCT performed in moderate and severe/very severe COPD patients, combining formoterol (FOR) 12 μg BID with TIO 18 μg QD was more effective at improving the area under the curve (AUC) for FEV_1_ measured 0–4 h post morning dose (FEV_1_ AUC_0-4 h_) in both men (+ 410 mL) and women (+ 320 mL) than administering TIO alone (+ 190 mL and + 180 mL, respectively in men and women; P < 0.01). In women, the mean percentage change in FEV_1_ AUC_0-4 h_ was in the range of 31.7–34.7% with FOR/TIO vs. 18.5–20.9% with TIO [49]. Men showed comparable ranges to those in women with FOR/TIO (32.9–35.7%) and TIO (15.7–19.7%) [49].

In a pooled analysis [50] of six parallel-group studies included in the IGNITE program, 26 weeks of treatment with indacaterol/GLY (IND/GLY) 100/50 μg QD improved trough FEV_1_ in both men and women with moderate to very severe and severe to very severe COPD vs. FP/SAL 500/50 μg, GLY 50 μg, TIO 18 μg, and PCB (P < 0.01). Men treated with IND/GLY vs. FP/SAL or PCB experienced greater improvements in trough FEV_1_ than women, while women administered IND/GLY vs. TIO had similarly higher improvements than men [50]. IND/GLY was superior to all comparators in terms of reduction in SGRQ and Transition Dyspnea Index (TDI) total scores, however it resulted more effective in women than men [50]. The use of rescue medications and symptoms total score were numerically lower in women than men after treatment with IND/GLY vs. all comparators [50].

In a pooled analysis of the Phase III ACLIFORM and AUGMENT RCTs performed in moderate to severe COPD patients [51], 24 weeks of treatment with aclidinium (ACL)/FOR 400/12 μg BID improved trough FEV_1_ in both men and women (+ 163 mL and + 101 mL, respectively; P < 0.001 vs. PCB) and post-dose FEV_1_ in men and women (+ 334 mL and + 231 mL, respectively; P < 0.001 vs. PCB). In men, ACL/FOR was superior to ACL and FOR monotherapies on trough FEV_1_ (+ 44 mL and + 86 mL, respectively; P < 0.01) and post-dose FEV_1_ (+ 148 mL and + 125 mL, respectively; P < 0.001) [51]. Women treated with ACL/FOR experienced an improvement in trough FEV_1_ vs. FOR (+ 41 mL; P < 0.05) but not vs. ACL, whereas post-dose FEV_1_ was increased vs. FOR (+ 93 mL; P < 0.001) and ACL (+ 67 mL; P < 0.01) [51]. The effect of ACL/FOR on TDI focal score was greater than PCB in both men and women (+ 1.36 and + 1.54 units, respectively; P < 0.001) and in men the improvement of 0.54 units was significant (P < 0.05) vs. FOR [51]. A trend towards lower rates of moderate/severe exacerbations based on healthcare resource utilization were observed for ACL/FOR vs. PCB and vs. monotherapies in both men and women [51]. The reduction in the EXACT exacerbation rate per patient/year was significant (P < 0.01) for men treated with ACL/FOR vs. PCB (RR 0.71) [51].

A post-hoc analysis of the EFfect of Indacaterol Glycopyrronium Vs Fluticasone Salmeterol on COPD Exacerbations (FLAME) RCT [52] found that in men with moderate to severe COPD, 1-year treatment with IND/GLY 110/50 μg QD was superior to FP/SAL 500/50 μg BID in reducing the annualized rates of moderate/severe exacerbations and all exacerbations (RR 0.81, 95%CI 0.73–0.91 and RR 0.88, 95%CI 0.81–0.96, respectively; P < 0.01), whereas women experienced numerically higher improvements. Compared to FP/SAL, IND/GLY increased the time to first moderate/severe exacerbation in men (HR 0.79, 95%CI 0.70–0.89; P < 0.001) and women (HR 0.76, 95%CI 0.63–0.91; P < 0.01) and the time to first all exacerbations in men (HR 0.86, 95%CI 0.79–0.94; P < 0.01) and women (HR 0.80, 95%CI 0.69–0.93; P < 0.01) [52]. The improvement in trough FEV_1_ was greater with IND/GLY treatment vs. FP/SAL in both men and women (+ 67 mL, 95%CI 51–84 and + 42 mL, 95%CI 12–71, respectively; P < 0.01 [52]. IND/GLY reduced the SGRQ total score in men by -1.3 units (95%CI -2.3 – -0.4; P < 0.01 vs. FP/SAL) and a numerical improvement was seen in women [52]. The use of rescue medications was reduced more with IND/GLY than with FP/SAL in men (-0.27 puffs/day, 95%CI -0.43 – -0.12; P < 0.001), but only numerically in women [52].

#### PDE4 inhibitor

A pooled analysis of the Roflumilast in the Prevention of COPD Exacerbations While Taking Appropriate Combination Treatment (REACT) and the Roflumilast Effect on Exacerbations in Patients on Dual Therapy (RE^2^SPOND) RCTs [54] documented that the phosphodiesterase (PDE4) inhibitor roflumilast 500 μg QD reduced the rate of moderate to severe exacerbations in men with COPD (RR 0.82, 95%CI 0.73–0.93; P < 0.01 vs. PCB), while women showed only a numerical decrease after 1 year of therapy.

An analysis of data from trial reports and systematic reviews released by the US FDA [53] showed no sex-related differences in the net benefit-harm index estimated for the treatment with roflumilast 500 μg QD in moderate to severe COPD patients with a history of exacerbations.

#### Bronchoreversibility to short acting bronchodilators

The LHS of smoking patients with mild COPD [57] found that women were numerically more likely to have a 10.0% increase in post-bronchodilator FEV_1_ than men undergoing methacholine bronchoprovocation test.

An analysis of data from a selected cohort of the LHS characterizing long-term changes in acute bronchodilator response to isoproterenol 200 μg over 11 years [59] found that relative and FEV_1_% predicted responses were not affected by sex differences, although absolute response was greater in men than women (127.3 mL and 86.6 mL, respectively; P < 0.001).

The population-based Proyecto Latinoamericano de Investigación en Obstrucción Pulmonar (PLATINO) study [58] documented that acute bronchodilator reversibility to SALB was more common in women affected by COPD than in men (32.9% and 23.9%, respectively; P < 0.01).

#### Antibiotics

One year of treatment with azithromycin 250 mg QD in addition to usual inhaled therapy reduced the frequency of exacerbations in COPD patients at increased risk of exacerbations, regardless of sex (P < 0.05 vs. PCB) [55].

Han et al. [56] documented that when adjusted for relevant confounders, adding azithromycin 250 mg QD to usual care for one year improved the time to first exacerbation in both men (HR 0.72, 95%CI 0.59–0.89; P < 0.01 vs. PCB) and women (HR 0.69, 95%CI 0.55–0.87; P < 0.01 vs. PCB).

### Nicotinic acetylcholine receptor partial agonist

In a multicentre RCT [60], mild to moderate COPD patients receiving varenicline for 12 weeks achieved a superior abstinence rate from smoking compared to PCB regardeless of sex (OR 8.57, 95%CI 4.55–16.2 and OR 6.27, 95%CI 2.71–14.5, respectively in men and women; P < 0.05).

### Studies including a mixed asthma and COPD population

A study conducted in primary care settings on a mixed population including asthmatic (10.6%) and COPD patients (3.5%) [62] found that bronchoreversibility response to SALB was numerically greater in men than women with mild obstruction, but no sex-related differences were detected when the obstruction was moderate or severe.

### Evidence synthesis

**Fig. 2:**
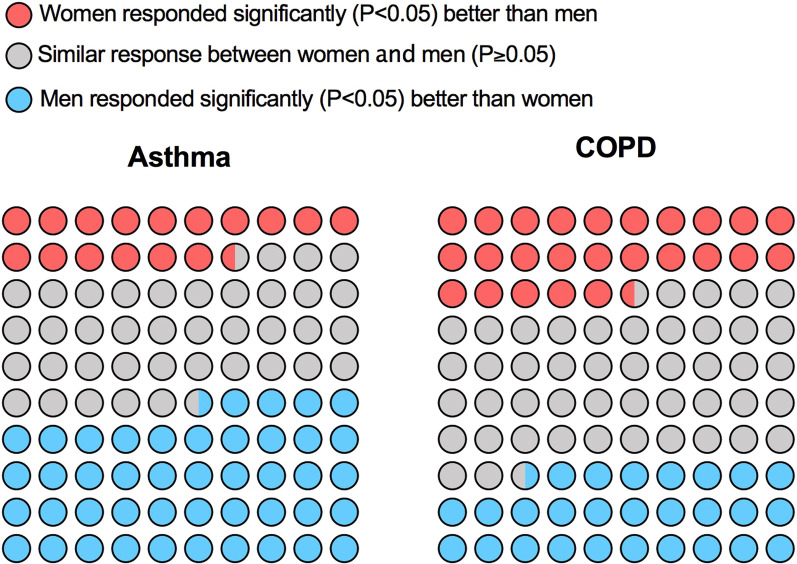
Parts of Whole graph (10 × 10 dot plot) reporting the amount of evidence concerning the impact of sex on the response to the overall pharmacological treatments for asthma and COPD resulting from the studies included in the systematic review. The greater response of a gender vs. the other one was reported when a statistically significant (P < 0.05) superiority was detected for any outcome. *COPD* chronic obstructive pulmonary disease

In asthma 44% of the evidence reported that men responded better than women to the treatments included in this systematic review, whereas this percentage was 28% in COPD. Less evidence supported a greater response of women than men to the therapy of asthma and COPD, namely in 17% and 26% respectively. Detailed information on the impact of sex on the response to the overall pharmacological treatments resulting from this systematic review in asthma and COPD is shown in Fig. 2.

**Fig. 3:**
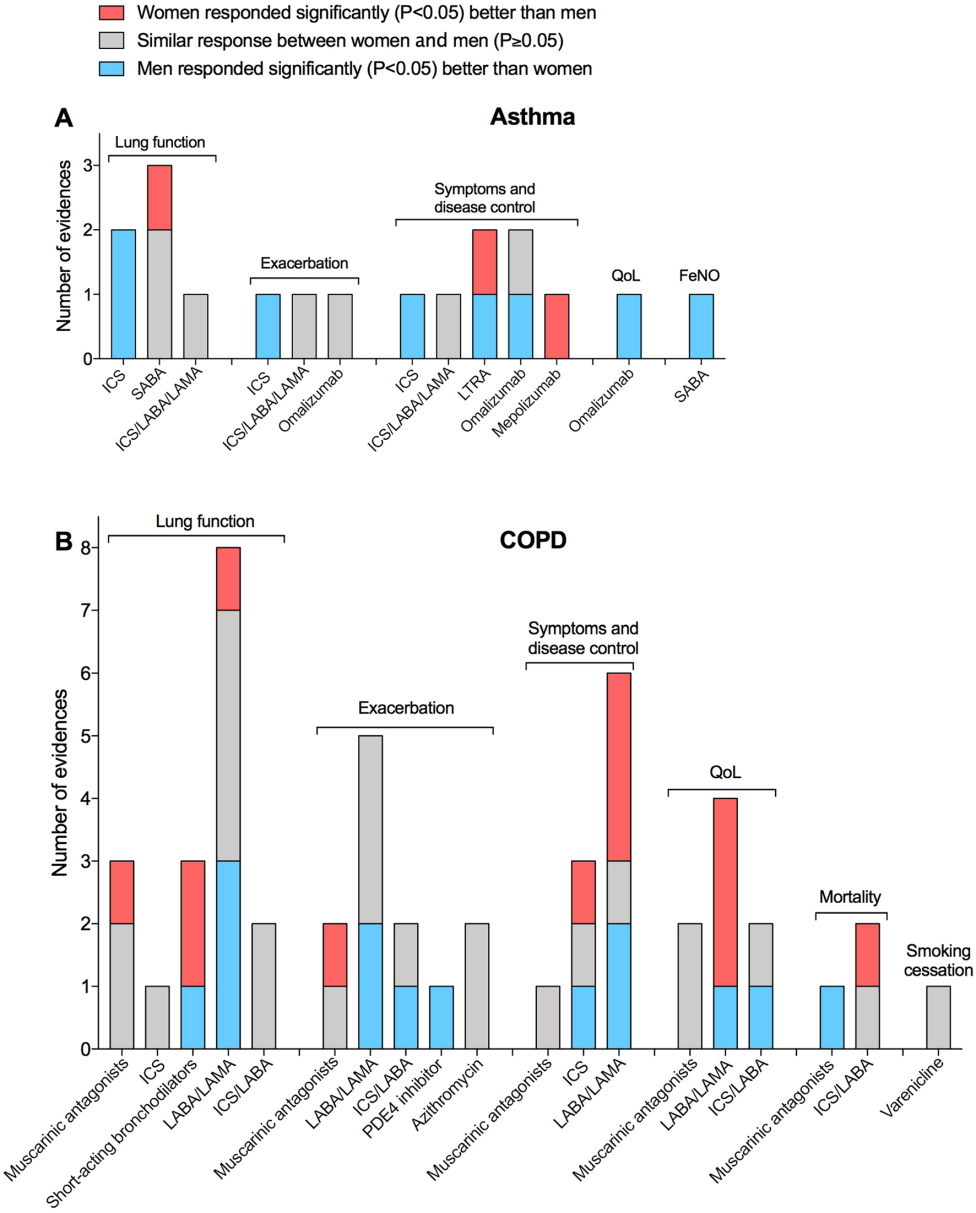
Response to pharmacological treatments in asthma **A** and COPD **B** according to specific outcomes and number of evidences as resulting from the studies included in the systematic review. The greater response of a gender vs. the other one was reported when a statistically significant (P < 0.05) superiority was detected for a specific treatment. *COPD* chronic obstructive pulmonary disease, *FeNO* exhaled nitric oxide, *ICS* inhaled corticosteroids, *LABA* long-acting β_2_-adrenoceptor agonist, *LAMA* long-acting muscarinic antagonist, *PDE4* phosphodiesterase 4, *SABA* short-acting β_2_-adrenoceptor agonist

Less evidence is currently available for asthma than in COPD concerning the role of sex on the efficacy of therapy, with detailed information on specific treatments and outcomes reported in Fig. 3A, B.

### Risk of bias and quality of evidence

**Fig. 4:**
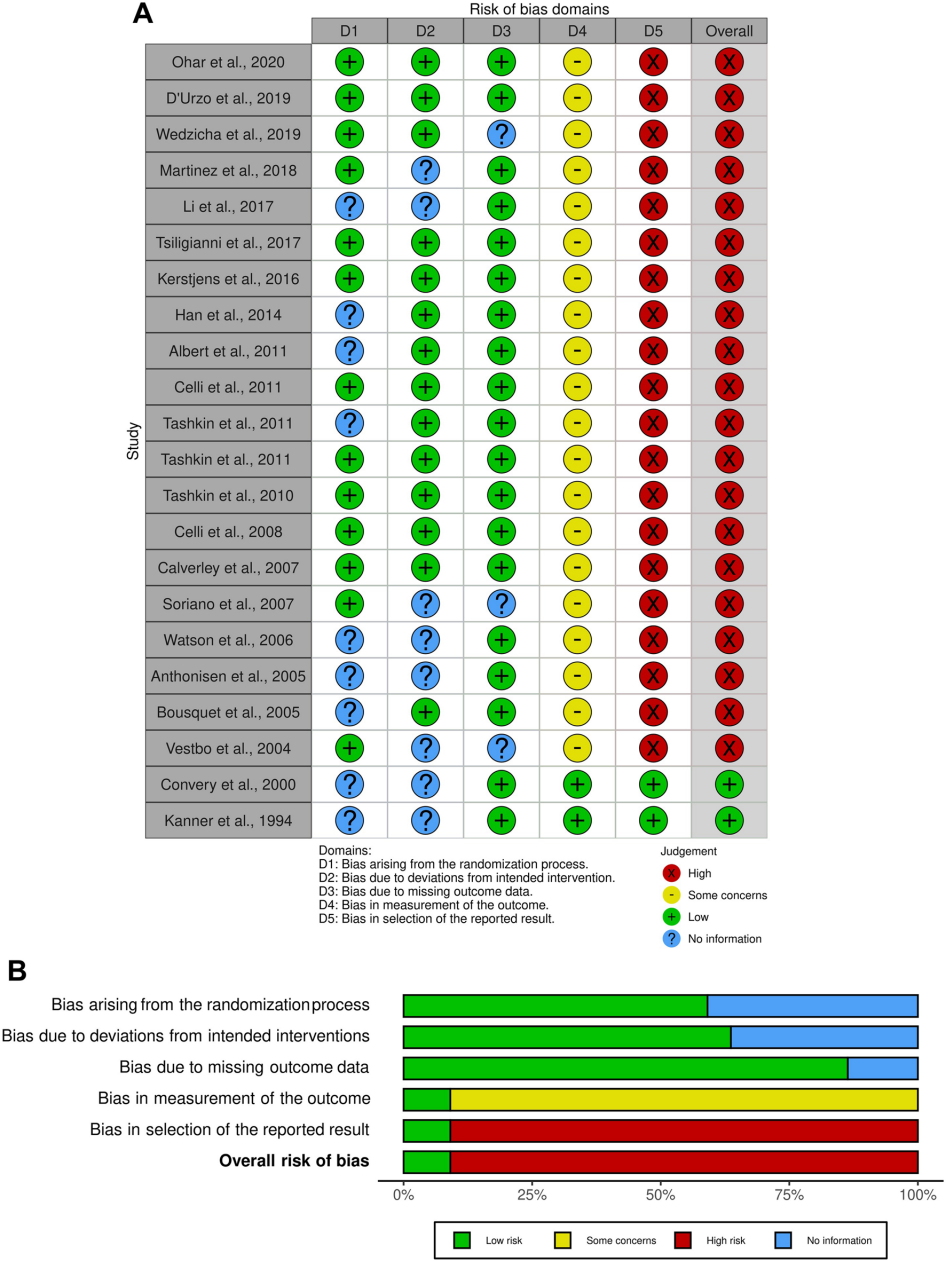
Traffic light plot for the assessment of the risk of bias of each included randomized trial **A** and weighted plot for the assessment of the overall risk of bias **B** via the Cochrane RoB 2 tool (B) (n = 22 studies). Traffic light plot reports five risk of bias domains: D1, bias arising from the randomization process; D2, bias due to deviations from intended intervention; D3, bias due to missing outcome data; D4, bias in measurement of the outcome; D5, bias in selection of the reported result; green circle represents low risk of bias, yellow circle indicates some concerns on the risk of bias, red circle reports high risk of bias, and blue circle indicates insufficient information on the risk of bias. *RoB* risk of bias

Of the 22 trials assessable via the Cochrane RoB 2 [31, 34, 39, 41–52, 54–57, 59–61], a low risk of bias was reported in 13 studies (59.1%) for randomization process, in 14 studies (63.6%) for deviations from intended interventions, in 19 studies (86.4%) for missing outcome data. Some studies did not report information for the risk of bias in the randomization process (9, 40.9%), deviations from intended interventions (8, 36.4%), and missing outcome data (3, 13.6%). Most the studies (20, 90.9%) had some concerns on the risk of bias for the measurement of the outcomes and a high risk of bias for the selection of the reported results. The overall risk of bias was high for most studies (20, 90.9%). Detailed information concerning the risk of bias assessment is reported in Fig. 4. Almost all the included randomized studies were ranked as being of medium- to high-quality according to Jadad score (Table 1).

The overall quality of evidence from the observational cohort and cross-sectional studies, assessed respectively via the NOS score and JBI Checklist tool, is presented in Table 1. Two cohort studies [38, 40] were given a NOS score ≥ 7 and were considered of high quality, whereas four studies [33, 36, 37] were assigned a score of ≤ 6. Quality assessment of cross-sectional studies indicated that one study [58] was at high risk of bias for the evaluated outcomes, another one [62] was at medium risk, and the study performed by Siroux et al. [32] was at low risk of bias.

## Discussion

The findings resulting from this systematic review indicate that the effectiveness of therapy for asthma and COPD may be modulated by sex. When considering relevant outcomes such as lung function, exacerbation, symptoms and disease control, the current evidence is generally conflicting, although some consistent data could be found especially in asthma.

For instance, in asthmatic patients ICS was more effective in men than women in improving lung function, symptoms and disease control, and in preventing exacerbation; conversely, ICS/LABA/LAMA combination was equally effective in both men and women in improving lung function and disease control, and in reducing exacerbation. Unexpectedly, no studies are currently available on the impact of sex on ICS/LABA combination in asthma. Conflicting data are available for the effect of sex on the effectiveness of mAbs in asthmatic patients.

Regarding COPD, the current evidence is much more heterogeneous. ICS and ICS/LABA combination resulted equally effective in men and women on lung function; concerning exacerbation, PDE4 inhibitor was more effective in men than women, whereas azithromycin was equally effective in both sexes; no sex-related difference was detected for muscarinic antagonists on disease control. Considering the dual bronchodilation therapy, men responded better than women when LABA/LAMA was compared to ICS/LABA against the risk of exacerbation, whereas no sex-related influence was detected vs. LAMA and LABA. Dyspnea improved more in women than in men when comparing LABA/LAMA vs. ICS/LABA and LAMA, but not vs. LABA. Inconsistent data are available for the impact of sex on the effect of LABA/LAMA vs. ICS/LABA and LAMA on lung function. Surprisingly, no studies have been performed to assess the sex-related response to ICS/LABA/LAMA in COPD.

Several studies have investigated the role of sex on receptor expression in human airways and murine models of chronic obstructive respiratory disorders. As expected, the main evidence was raised from research on sex-steroids that activate estrogen receptors (ER) or androgen receptors (AR).

AR signalling induced by androgens stabilizes CD4^+^ regulatory T cells (Tregs) suppressive function, providing a mechanism for higher prevalence of asthma in women compared with men [64]. This evidence is supported by the fact that the higher airway expression of AR and higher androgen levels in men are associated with better lung function, fewer symptoms, and a lower fraction exhaled nitric oxide (FeNO) in asthma [65]. Furthermore, the activation of AR may exert beneficial effect in asthma by ameliorating airway hyperresponsiveness (AHR) and type 2 inflammation via reducing intracellular calcium influx and modulating complex mechanisms such as the interleukin (IL) 17A pathway [66–68].

Concerning estrogens, they mainly act by activating both the ER forms, with ER-α having detrimental effect in the airways and ER-β being characterized by protective activity against AHR and remodelling. These beneficial effects are mediated by the reduction of intracellular calcium, suppression of nuclear factor kappa B (NF-κB) pathway, and modulation of platelet-derived growth factor (PDGF) inducing airway smooth muscle (ASM) proliferation [69–73]. The variations in ERα and ERβ expression profile on ASM during inflammation may contribute to estrogen signaling in asthma [74]. In addition, estrogens may enhance the IL-4–induced M2 gene expression in alveolar macrophages and those derived from bone marrow [75]. Thus, an imbalance in the expression or activity of ERα and ERβ may be linked to the severity of disease in women.

Also other sex hormones, such as progesterone (P4), may have a role in asthma by altering the function of a key component of the mucociliary apparatus [76]. Furthermore, while normal women have cyclical changes in the function and density of β_2_-AR in the luteal phase during the premenstrual period, in asthmatic patients a loss of the normal cyclical pattern in β_2_-AR regulation has been detected, a condition related to AHR during bronchoprovocation test [77].

Sex hormones may modulate also the expression of muscarinic acetylcholine receptors (mAChR). The activation of ER-α is related to altered expression of M_2_ mAChR, leading to increased AHR [78]. Moreover, in women with COPD the lungs have a greater gene expression for the M_3_ mAChR relative to M_2_ mAChR than in male [44]. Of note, the extent of bronchorelaxant response is related with BMI, such that a larger improvement in lung function elicited by muscarinic antagonists has been reported in thin women [44].

Indeed, COPD is characterized by high sex-dependent T-cell profile. In this regard, a greater expression of chemokine receptor CCR5 on CD8^+^ T cells and higher amount of CXCR3^+^CD8^+^ T cells was detected in bronchoalveolar lavage (BAL) or blood in women smokers with COPD compared to those without COPD. Moreover, across these patients the Th1/Tc1 immune response was related to macrophage count in BAL and goblet cell density, and the extent of emphysema was associated to the Th2/Tc2 response [44]. Conversely, the expression of CCR5 on CD4^+^ and CD8^+^ T cells was lower in BAL from male smokers with COPD compared to subjects without COPD [79]. Overall, this evidence supports different links between cellular events, inflammation, and clinical manifestations of COPD in women compared to men.

Most of the pre-clinical evidence regarding the influence of sex on the expression of receptors in the airways originate from murine models of AHR that, unfortunately, may have just a relative translational impact on the pharmacotherapy in asthma and COPD. Moreover, across the records included in this systematic review, only 2 RCTs were specifically designed to assess the influence of sex on the effectiveness of treatment in asthma [31] and COPD [57]. The remaining papers reported data from trials or post-hoc analyses of previous studies for which the assessment of sex on asthma and COPD therapy was not even a pre-specified endpoint, leading to high risk of Type I error, or observational trials that were characterized by major intrinsic limitations. Another limitation of this systematic review is related to the unbalanced number of males and females enrolled in the studies, especially in COPD: almost all the trials had a higher number of males than females. Thus, the high risk of bias resulting from the Cochrane RoB 2 tool was extensively expected, suggesting that the provided evidence should be interpreted with caution.

## Conclusions

Indeed, the findings of this systematic review highlight that the number of studies in asthma and COPD looking at the same drug and outcome is currently small, making difficult to draw solid conclusions. However it seems that, as supported also by pre-clinical findings, ICS may be generally less effective in women than in men to treat asthma. Consistent evidence also suggests that in asthmatic patients ICS/LABA/LAMA combination may be equally effective in both men and women. Overall, excluding the effort of independent research, Big Pharma has demonstrated scarce interest in assessing the potential different impact of sex on the pharmacological response to asthma and COPD therapy. In this regard, this systematic review highlights the strong pharmacological and clinical need of adequately investigating this issue that to date remains very controversial. A first step to manage this important and discriminatory scientific lack could be to make the data from large investigational clinical trials in asthma and COPD available specifically for each sex rather than as overall results. Moreover, considering that clinical trials in asthma and COPD are characterized by imbalanced enrollment ratio between men and women leading to possible sex bias in measured outcomes [9, 80], it is expected that the randomization procedures of future RCTs will be set to equally enroll both sexes. Finally, but not less important, pre-specified analyses in men and women should be planned in the trial protocols, a necessary condition that should be requested also by the regulatory agencies.

## Data Availability

All data generated or analysed during this study are included in this published article.

## Abbreviations

ACQ: Asthma Control Questionnaire
AHR: Airway hyperresponsiveness
ASM: Airway smooth muscle
AR: Androgen receptor
AUC: Area under the curve
β_2_-AR: β_2_-Adrenoceptor
BAL: Bronchoalveolar lavage
BD: Bronchodilator
BID: Twice daily
B-IPQ: Brief Illness Perception Questionnaire
BMI: Body mass index
CENTRAL: Cochrane Central Register of Controlled Trials
COPD: Chronic obstructive pulmonary disease
DECiMAL: Data extraction for complex meta-analysis
ER: Estrogen receptor
EXACT: Exacerbation of Chronic Pulmonary Disease Tool
EXACT-RS: EXACT-respiratory symptoms
FDA: Food and Drug Administration
FeNO: Fraction exhaled nitric oxide
FEV_1_: Forced expiratory volume in the 1st second
FEV_1_ AUC_0-4__ h_: FEV_1_ measured 0–4 h post morning dose
FOR: Formoterol
FP: Fluticasone propionate
GLY: Glycopyrronium bromide
HR: Hazard ratio
IB: Ipratropium bromide
ICS: Inhaled corticosteroid
IL: Interleukin
IND: Indacaterol
JBI: Joanna Briggs Institute
LABA: Long-acting β_2_-AR agonist
LAMA: Long-acting muscarinic antagonist
mAChR: Muscarinic acetylcholine receptor
NCD: Non-communicable disease
NF-κB: Nuclear factor kappa B
NOS: Newcastle–Ottawa Scale
OCS: Oral corticosteroid
OR: Odds ratio
PCB: Placebo
PD: Pharmacodynamics
PDE4: Phosphodiesterase 4
PDGF: Platelet-derived growth factor
PD_20_: Provocative dose of methacholine causing a 20% fall in FEV_1_
QD: Once daily
PRISMA-P: Preferred Reporting Items for Systematic Reviews and Meta-Analyses Protocols
RCT: Randomized controlled trial
RoB 2: Risk of Bias 2
RR: Relative rate
SALB: Salbutamol
SAL: Salmeterol
SGRQ: St George's Respiratory Questionnaire
Tregs: Regulatory T cells
TDI: Transition Dyspnea Index
TIO: Tiotropium

## References

[1] GINA. 2021 GINA Main Report|Global Initiative for Asthma [Internet]. https://ginasthma.org/wp-content/uploads/2021/05/GINA-Main-Report-2021-V2-WMS.pdf. Accessed 22 Jul 2022.

[2] 2021 GOLD Reports - Global Initiative for Chronic Obstructive Lung Disease—GOLD [Internet]. https://goldcopd.org/2021-gold-reports/. Accessed 10 Mar 2022.

[3] Chronic obstructive pulmonary disease (COPD) [Internet]. https://www.who.int/news-room/fact-sheets/detail/chronic-obstructive-pulmonary-disease-(copd). Accessed 22 Feb 2022.

[4] Asthma [Internet]. https://www.who.int/news-room/fact-sheets/detail/asthma. Accessed 22 Feb 2022.

[5] Roche N, Plaza V, Backer V, van der Palen J, Cerveri I, Gonzalez C (2020). Asthma control and COPD symptom burden in patients using fixed-dose combination inhalers (SPRINT study). NPJ Prim care Respir Med..

[6] Chowdhury NU, Guntur VP, Newcomb DC, Wechsler ME (2021). Sex and gender in asthma. Eur Respir Rev.

[7] Calzetta L, Puxeddu E, Rogliani P (2017). Gender-related responsiveness to the pharmacological treatment of COPD: a first step towards the personalized medicine. EBioMedicine.

[8] Mauvais-Jarvis F, Bairey Merz N, Barnes PJ, Brinton RD, Carrero JJ, DeMeo DL (2020). Sex and gender: modifiers of health, disease, and medicine. Lancet.

[9] Jenkins CR, Chapman KR, Donohue JF, Roche N, Tsiligianni I, Han MLK (2017). Improving the management of COPD in women. Chest.

[10] Jenkins CR, Boulet L-P, Lavoie KL, Raherison-Semjen C, Singh D (2022). Personalized treatment of asthma: the importance of sex and gender differences. J allergy Clin Immunol Pract..

[11] Alexander J, Edwards RA, Savoldelli A, Manca L, Grugni R, Emir B (2017). Integrating data from randomized controlled trials and observational studies to predict the response to pregabalin in patients with painful diabetic peripheral neuropathy. BMC Med Res Methodol..

[12] Arditi C, Burnand B, Peytremann-Bridevaux I (2016). Adding non-randomised studies to a Cochrane review brings complementary information for healthcare stakeholders: an augmented systematic review and meta-analysis. BMC Health Serv Res.

[13] Shrier I, Boivin JF, Steele RJ, Platt RW, Furlan A, Kakuma R (2007). Should meta-analyses of interventions include observational studies in addition to randomized controlled trials? A critical examination of underlying principles. Am J Epidemiol.

[14] Norris S, Atkins D, Bruening W, Fox S, Johnson E, Kane R, et al. Selecting observational studies for comparing medical interventions. methods guide for effectiveness and comparative effectiveness reviews. 2008. 21433401

[15] Gershon AS, Jafarzadeh SR, Wilson KC, Walkey AJ (2018). Clinical knowledge from observational studies: everything you wanted to know but were afraid to ask. Am J Respir Crit Care Med.

[16] Moher D, Shamseer L, Clarke M, Ghersi D, Liberati A, Petticrew M (2015). Preferred reporting items for systematic review and meta-analysis protocols (PRISMA-P) 2015 statement. Syst Rev.

[17] Schardt C, Adams MB, Owens T, Keitz S, Fontelo P (2007). Utilization of the PICO framework to improve searching PubMed for clinical questions. BMC Med Inf Decis Mak.

[18] Somayaji R, Chalmers JD, Weatherald J, Humbert M, Riha R (2022). Just breathe: a review of sex and gender in chronic lung disease. Eur Respir Rev.

[19] Matera MG, Ora J, Calzetta L, Rogliani P, Cazzola M (2021). Sex differences in COPD management. Expert Rev Clin Pharmacol.

[20] Barbagelata E, Nicolini A, Ambrosino I, Politi C (2018). Gender differences and Chronic obstructive pulmonary disease: an update on the literature. Ital J Med.

[21] Gut-Gobert C, Cavaillès A, Dixmier A, Guillot S, Jouneau S, Leroyer C (2019). Women and COPD: Do we need more evidence?. Eur Respir Rev.

[22] Hunninghake GM, Gold DR (2009). Sexual dimorphism: is it relevant to steroid resistance or asthma control?. J Allergy Clin Immunol.

[23] Jadad AR, Moore RA, Carroll D, Jenkinson C, Reynolds DJ, Gavaghan DJ (1996). Assessing the quality of reports of randomized clinical trials: is blinding necessary?. Control Clin Trials.

[24] Higgins JPT, Savović J, Page MJ, Elbers RG, Sterne JAC. Chapter 8: Assessing risk of bias in a randomized trial. Cochrane Handbook for Systematic Reviews of Interventions version 6.0 (updated July 2019). Cochrane, 2019. www.training.cochrane.org/handbook. 2019;205–28.

[25] GA Wells D O’Connell, J Peterson, V Welch, M Losos, P Tugwell, BS. The Newcastle-Ottawa Scale (NOS) for assessing the quality of nonrandomised studies in meta-analyses. 2020. 2014.

[26] Moola S, Munn Z, Tufanaru C, Aromataris E, Sears K, Sfetcu R, et al. Chapter 7: Systematic reviews of etiology and risk. In: Aromataris E, Munn Z, editors. JBI Manual for Evidence Synthesis. 2020.

[27] Pedder H, Sarri G, Keeney E, Nunes V, Dias S (2016). Data extraction for complex meta-analysis (DECiMAL) guide. Syst Rev.

[28] Sterne JAC, Savovic J, Page MJ, Elbers RG, Blencowe NS, Boutron I (2019). RoB 2: a revised tool for assessing risk of bias in randomised trials. BMJ.

[29] McGuinness LA (2019). robvis: an R package and web application for visualising risk-of-bias assessments. Res Synth Methods.

[30] Rogliani P, Beasley R, Cazzola M, Calzetta L (2021). SMART for the treatment of asthma: a network meta-analysis of real-world evidence. Respir Med.

[31] Convery RP, Leitch DN, Bromly C, Ward RJ, Bartlett G, Hendrick DJ (2000). Effect of inhaled fluticasone propionate on airway responsiveness in treatment-naive individuals - a lesser benefit in females. Eur Respir J.

[32] Siroux V, Boudier A, Bousquet J, Bresson JL, Cracowski JL, Ferran J (2009). Phenotypic determinants of uncontrolled asthma. J Allergy Clin Immunol.

[33] Dijkstra A, Vonk JM, Jongepier H, Koppelman GH, Schouten JP, Ten Hacken NHT (2006). Lung function decline in asthma: association with inhaled corticosteroids, smoking and sex. Thorax.

[34] Kerstjens HAM, Moroni-Zentgraf P, Tashkin DP, Dahl R, Paggiaro P, Vandewalker M (2016). Tiotropium improves lung function, exacerbation rate, and asthma control, independent of baseline characteristics including age, degree of airway obstruction, and allergic status. Respir Med.

[35] Lima JJ, Mohamed MHN, Self TH, Eberle LV, Johnson JA (2000). Importance of beta(2)adrenergic receptor genotype, gender and race on albuterol-evoked bronchodilation in asthmatics. Pulm Pharmacol Ther.

[36] Nerpin E, Ferreira DS, Weyler J, Schlunnsen V, Jogi R, RaherisonSemjen C (2021). Bronchodilator response and lung function decline: associations with exhaled nitric oxide with regard to sex and smoking status. World Allergy Organ J.

[37] Colombo D, Zagni E, Ferri F, Canonica GW, Astarita C, Balbo P (2019). Gender differences in asthma perception and its impact on quality of life: a post hoc analysis of the PROXIMA (Patient Reported Outcomes and Xolair® in the Management of Asthma) study. Allergy Asthma Clin Immunol.

[38] Harvey ES, Langton D, Katelaris C, Stevens S, Farah CS, Gillman A (2020). Mepolizumab effectiveness and identification of super-responders in severe asthma. Eur Respir J.

[39] Bousquet J, Cabrera P, Berkman N, Buhl R, Holgate S, Wenzel S (2005). The effect of treatment with omalizumab, an anti-IgE antibody, on asthma exacerbations and emergency medical visits in patients with severe persistent asthma. Allergy Eur J Allergy Clin Immunol.

[40] Schermer TRJ, Hendriks AJC, Chavannes NH, Dekhuijzen PNR, Wouters EFM, Van Den Hoogen H (2004). Probability and determinants of relapse after discontinuation of inhaled corticosteroids in patients with COPD treated in general practice. Prim Care Respir J.

[41] Watson L, Schouten JP, Löfdahl CG, Pride NB, Laitinen LA, Postma DS (2006). Predictors of COPD symptoms: does the sex of the patient matter?. Eur Respir J.

[42] Soriano JB, Sin DD, Zhang X, Camp PG, Anderson JA, Anthonisen NR (2007). A pooled analysis of FEV1 decline in COPD patients randomized to inhaled corticosteroids or placebo. Chest.

[43] Tashkin D, Celli B, Kesten S, Lystig T, Decramer M (2010). Effect of tiotropium in men and women with COPD: results of the 4-year UPLIFT® trial. Respir Med.

[44] Li X, Obeidat M, Zhou G, Leung JM, Tashkin D, Wise R (2017). Responsiveness to ipratropium bromide in male and female patients with mild to moderate chronic obstructive pulmonary disease. EBioMedicine.

[45] Ohar JA, Ozol-Godfrey A, Goodin T, Sanjar S (2020). Effect of gender on lung function and patient-reported outcomes in patients with copd receiving nebulized glycopyrrolate. Int J COPD.

[46] Calverley PMA, Anderson JA, Celli B, Ferguson GT, Jenkins C, Jones PW (2007). Salmeterol and fluticasone propionate and survival in chronic obstructive pulmonary disease. N Engl J Med..

[47] Vestbo J, Soriano JB, Anderson JA, Calverley P, Pauwels R, Jones P (2004). Gender does not influence the response to the combination of salmeterol and fluticasone propionate in COPD. Respir Med.

[48] Celli BR, Thomas NE, Anderson JA, Ferguson GT, Jenkins CR, Jones PW (2008). Effect of pharmacotherapy on rate of decline of lung function in chronic obstructive pulmonary disease: Results from the TORCH study. Am J Respir Crit Care Med.

[49] Tashkin DP, Varghese ST (2011). Combined treatment with formoterol and tiotropium is more efficacious than treatment with tiotropium alone in patients with chronic obstructive pulmonary disease, regardless of smoking status, inhaled corticosteroid use, baseline severity, or gender. Pulm Pharmacol Ther.

[50] Tsiligianni I, Mezzi K, Fucile S, Kostikas K, Shen S, Banerji D (2017). Response to Indacaterol/Glycopyrronium (IND/GLY) by sex in patients with COPD: a pooled analysis from the IGNITE program. COPD J Chronic Obstr Pulm Dis.

[51] D’Urzo AD, Singh D, Donohue JF, Kerwin EM, Ribera A, Molins E (2019). Efficacy of aclidinium/formoterol 400/12 µg, analyzed by airflow obstruction severity, age, sex, and exacerbation history: pooled analysis of ACLIFORM and AUGMENT. Int J COPD.

[52] Wedzicha JA, Singh D, Tsiligianni I, Jenkins C, Fucile S, Fogel R (2019). Treatment response to indacaterol/glycopyrronium versus salmeterol/fluticasone in exacerbating COPD patients by gender: a post-hoc analysis in the FLAME study 11 Medical and Health Sciences 1102 Cardiorespiratory Medicine and Haematology. Respir Res.

[53] Yu T, Fain K, Boyd CM, Singh S, Weiss CO, Li T (2014). Benefits and harms of roflumilast in moderate to severe COPD. Thorax.

[54] Martinez FJ, Rabe KF, Calverley PMA, Fabbri LM, Sethi S, Pizzichini E (2018). Determinants of response to roflumilast in severe chronic obstructive pulmonary disease: pooled analysis of two randomized trials. Am J Respir Crit Care Med.

[55] Albert RK, Connett J, Bailey WC, Casaburi R, Cooper JAD, Criner GJ (2011). Azithromycin for prevention of exacerbations of COPD. N Engl J Med.

[56] Han MLK, Tayob N, Murray S, Dransfield MT, Washko G, Scanlon PD (2014). Predictors of chronic obstructive pulmonary disease exacerbation reduction in response to daily azithromycin therapy. Am J Respir Crit Care Med.

[57] Kanner RE, Connett JE, Altose MD, Buist AS, Lee WW, Tashkin DP (1994). Gender difference in airway hyperresponsiveness in smokers with mild COPD: the lung health study. Am J Respir Crit Care Med.

[58] Lopez Varela MV, Montes De Oca M, Halbert RJ, Muiño A, Perez-Padilla R, Tálamo C, et al. Sex-related differences in COPD in five Latin American cities: the PLATINO study. Eur Respir J 2010;36(5):1034–41. 10.1183/09031936.00165409 20378599

[59] Anthonisen NR, Lindgren PG, Tashkin DP, Kanner RE, Scanlon PD, Connett JE (2005). Bronchodilator response in the lung health study over 11 yrs. Eur Respir J.

[60] Tashkin DP, Rennard S, Hays JT, Ma W, Lawrence D, Lee TC (2011). Effects of varenicline on smoking cessation in patients with mild to moderate COPD: a randomized controlled trial. Chest.

[61] Celli B, Vestbo J, Jenkins CR, Jones PW, Ferguson GT, Calverley PMA (2011). Sex differences in mortality and clinical expressions of patients with chronic obstructive pulmonary disease: the TORCH experience. Am J Respir Crit Care Med.

[62] Dales RE, Mehdizadeh A, Aaron SD, Vandemheen KL, Clinch J (2006). Sex differences in the clinical presentation and management of airflow obstruction. Eur Respir J.

[63] Calzetta L, Aiello M, Frizzelli A, Bertorelli G, Ritondo BL, Rogliani P (2021). The impact of monoclonal antibodies on airway smooth muscle contractility in asthma: a systematic review. Biomedicines..

[64] Gandhi VD, Cephus J-Y, Norlander AE, Chowdhury NU, Zhang J, Ceneviva ZJ (2022). Androgen receptor signaling promotes Treg suppressive function during allergic airway inflammation. J Clin Invest.

[65] Zein JG, McManus JM, Sharifi N, Erzurum SC, Marozkina N, Lahm T (2021). Benefits of airway androgen receptor expression in human asthma. Am J Respir Crit Care Med.

[66] Kalidhindi RSR, Ambhore NS, Balraj P, Schmidt T, Khan MN, Sathish V (2021). Androgen receptor activation alleviates airway hyperresponsiveness, inflammation, and remodeling in a murine model of asthma. Am J Physiol Lung Cell Mol Physiol..

[67] Kalidhindi RSR, Katragadda R, Beauchamp KL, Pabelick CM, Prakash YS, Sathish V (2019). Androgen receptor-mediated regulation of intracellular calcium in human airway smooth muscle cells. Cell Physiol Biochem..

[68] Fuseini H, Yung JA, Cephus JY, Zhang J, Goleniewska K, Polosukhin VV (2018). Testosterone decreases house dust mite-induced Type 2 and IL-17A-mediated airway inflammation. J Immunol.

[69] Kalidhindi RSR, Ambhore NS, Bhallamudi S, Loganathan J, Sathish V (2020). Role of estrogen receptors α and β in a murine model of asthma: exacerbated airway hyperresponsiveness and remodeling in ERβ knockout mice. Front Pharmacol..

[70] Ambhore NS, Kalidhindi RSR, Pabelick CM, Hawse JR, Prakash YS, Sathish V (2019). Differential estrogen-receptor activation regulates extracellular matrix deposition in human airway smooth muscle remodeling via NF-κB pathway. FASEB J.

[71] Bhallamudi S, Connell J, Pabelick CM, Prakash YS, Sathish V (2020). Estrogen receptors differentially regulate intracellular calcium handling in human nonasthmatic and asthmatic airway smooth muscle cells. Am J Physiol Lung Cell Mol Physiol..

[72] Ambhore NS, Kalidhindi RSR, Loganathan J, Sathish V (2019). Role of differential estrogen receptor activation in airway hyperreactivity and remodeling in a murine model of asthma. Am J Respir Cell Mol Biol.

[73] Ambhore NS, Katragadda R, Raju Kalidhindi RS, Thompson MA, Pabelick CM, Prakash YS (2018). Estrogen receptor beta signaling inhibits PDGF induced human airway smooth muscle proliferation. Mol Cell Endocrinol.

[74] Aravamudan B, Goorhouse KJ, Unnikrishnan G, Thompson MA, Pabelick CM, Hawse JR (2017). Differential expression of estrogen receptor variants in response to inflammation signals in human airway smooth muscle. J Cell Physiol.

[75] Keselman A, Fang X, White PB, Heller NM (2017). Estrogen signaling contributes to sex differences in macrophage polarization during asthma. J Immunol.

[76] Jain R, Ray JM, Pan JH, Brody SL (2012). Sex hormone-dependent regulation of cilia beat frequency in airway epithelium. Am J Respir Cell Mol Biol.

[77] Tan KS, McFarlane LC, Lipworth BJ (1997). Loss of normal cyclical beta 2 adrenoceptor regulation and increased premenstrual responsiveness to adenosine monophosphate in stable female asthmatic patients. Thorax.

[78] Schmidt D, Dent G, Rühlmann E, Muñ oz N, Leff A, Rabe K (2002). Studying human airway pharmacology in microsections: application of videomicrometry. Eur Respir J.

[79] Forsslund H, Yang M, Mikko M, Karimi R, Nyrén S, Engvall B (2016). Gender differences in the T-cell profiles of the airways in COPD patients associated with clinical phenotypes. Int J Chron Obstruct Pulmon Dis..

[80] Ciudad-Gutiérrez P, Fernández-Rubio B, Guisado-Gil AB (2021). Gender bias in clinical trials of biological agents for severe asthma: a systematic review. PLoS ONE.

[81] Canonica GW, Ferrando M, Baiardini I, Puggioni F, Racca F, Passalacqua G (2018). Asthma: personalized and precision medicine. Curr Opin Allergy Clin Immunol.

[82] Sidhaye VK, Nishida K, Martinez FJ (2018). Precision medicine in COPD: where are we and where do we need to go?. Eur Respir Rev.

[83] Sin DD. Implementing COPD precision medicine in clinical practice. 2020. 10.1007/978-3-030-31507-8_25

